# Comparative Genome Analysis of *Psychrobacillus* Strain PB01, Isolated from an Iceberg

**DOI:** 10.4014/jmb.1909.09008

**Published:** 2019-12-09

**Authors:** Jun Young Choi, Sun Chang Kim, Pyung Cheon Lee

**Affiliations:** 1 Department of Molecular Science and Technology and Department of Applied Chemistry and Biological Engineering, Ajou University, Suwon 6499, Republic of Korea; 2 Department of Biological Sciences, Korea Advanced Institute of Science and Technology, Daejeon 34141, Republic of Korea

**Keywords:** *Psychrobacillus*, iceberg, TCA cycle, plasmid

## Abstract

A novel psychrotolerant *Psychrobacillus* strain PB01, isolated from an Antarctic iceberg, was comparatively analyzed with five related strains. The complete genome of strain PB01 consists of a single circular chromosome (4.3 Mb) and a plasmid (19 Kb). As potential low-temperature adaptation strategies, strain PB01 has four genes encoding cold-shock proteins, two genes encoding DEAD-box RNA helicases, and eight genes encoding transporters for glycine betaine, which can serve as a cryoprotectant, on the genome. The pan-genome structure of the six *Psychrobacillus* strains suggests that strain PB01 might have evolved to adapt to extreme environments by changing its genome content to gain higher capacity for DNA repair, translation, and membrane transport. Notably, strain PB01 possesses a complete TCA cycle consisting of eight enzymes as well as three additional *Helicobacter pylori*-type enzymes: ferredoxin-dependent 2-oxoglutarate synthase, succinyl-CoA/acetoacetyl-CoA transferase, and malate/quinone oxidoreductase. The co-existence of the genes for TCA cycle enzymes has also been identified in the other five *Psychrobacillus* strains.

## Introduction

Cold-adapted bacteria are classified into psychrophiles, which can grow at 0–20°C, or psychrotolerants (or psychrotrophs), which can grow at up to ~30°C [1]. Besides their biodiversity and ecological significance, cold-adapted bacteria have increasingly attracted attention due to their potential for biotechnological application, particularly in the use of cold-active enzymes and biomaterials in the food and chemical industries. A psychrotolerant strain, strain PB01, was isolated from an Antarctic iceberg near King Sejong Station. This strain is a pale yellow-colored, gram-positive rod-shaped bacterium belonging to the family *Bacillaceae* [2]. Three *Bacillus* strains were reclassified under the genus *Psychrobacillus*, based on polyphasic reexamination [2]. Currently, five strains of *Psychrobacillus* have been reported: *P. psychrotolerans* DSM 11706^T^ , *P. psychrodurans* DSM 11713^T^ , *P. insolitus* DSM 5^T^ , *P. soli* NHI-2^T^ , and P. lasiicapitis NEAU-3TGS17^T^ [3-6].

Moreover, the genomes of only five *Psychrobacillus* strains, namely *P. psychrotolerans*, *P. psychrodurans* DSM 11713, *Psychrobacillus* sp. FJAT-21963, *Psychrobacillus* sp. OK032, and *Psychrobacillus* sp. OK028, have been sequenced and deposited in public databases. However, the five genomes are not completely sequenced and thus do not contain sufficient data to provide the full genomic information of *Psychrobacillus* strains. The genome content and metabolic pathways of a *Psychrobacillus* strain whose genome is completely sequenced may provide a model strain for utilization in basic and biotechnological research. Furthermore, the genome-guided discovery of mobilomes, including plasmids, can accelerate the development of genetic engineering tools useful for manipulating *Psychrobacillus* strains. So far, however, there has been no report of a plasmid present in the five type strains of genus *Psychrobacillus*.

Here, we report the genomic characterization of the novel psychrotolerant strain PB01, which was isolated from an Antarctic iceberg, and provide a complete genome sequence of strain PB01. A comparative analysis of genomes of the five strains of *Psychrobacillus* was also described.

## Materials and Methods

### Genome Sequencing and Annotation

The genomic DNA of strain PB01 was extracted using the Genomic DNA Kit (Macrogen, Korea). The genome of strain PB01 was sequenced in SMRT cells using the Pacific Biosciences RS II single-molecule real-time sequencing technology kit (Pacific Biosciences, USA). Following sub-read filtering of raw read data via the PacBio RS II sequencer, 186,405 long reads and 1,658,531,999 base pairs, with a 386-fold genome coverage, were generated and de novo assembled using the Canu v1.3 assembler [7]. The circularization of contigs was performed using Circlator [8]. The open reading frames (ORFs) of one chromosome and one plasmid were predicted using the RAST server online [9]. tRNA and rRNA were predicted using tRNAscan-SE v1.21 [10], and RNAmmer v1.2 [11], respectively. Function predictions of the ORFs of the chromosome were made based on RPS-BLAST and BLASTp searches (e-value < 10^−3^) against the non-redundant GenBank protein database (www.ncbi.nlm.nih.gov/protein), the clusters of orthologous groups (COG) database (www.ncbi.nlm.nih.gov/COG), and the KEGG database (www.genome.jp/kegg). Graphical circular maps of the chromosome and the plasmid were produced using Circos v0.67 [12].

### Analysis of Average Nucleotide Identity (ANI)

The reference draft genome sequences of *P. psychrotolerans* (NZ_FOXU00000000.1), *P. psychrodurans* DSM 11713 (NZ_FOUN 00000000.1), *Psychrobacillus* sp. FJAT-21963 (NZ_LJIY00000000.1), *Psychrobacillus* sp. OK032 (NZ_FOGY00000000.1), and *Psychrobacillus* sp. OK028 (NZ_FNHY00000000.1) were downloaded from NCBI (www.ncbi.nlm.nih.gov/refseq). The draft genome contigs of the five strains were realigned against the complete genome of strain PB01 using Mauve Contig Mover [13]. The average nucleotide identity (ANI) values of strain PB01 and the five *Psychrobacillus* strains were analyzed using pyani v0.2.7 [14], a Python module, with default parameters. The synteny blocks (locally collinear blocks) of strain PB01 and the five *Psychrobacillus* strains were analyzed using progressiveMauve v.2.3.1 [15].

### Pan-Genome and Metabolic Pathway Analysis of *Psychrobacillus* Strains

The annotation files (GFF3) of strain PB01 and the five *Psychrobacillus* strains were generated using Prokka-1.12 [16] and were used for pan-genome analysis using Roary v3.11.2 [17] with a 70% minimum BLASTp percentage identity. The output files of Roary v3.11.2 were used to analyze and visualize the core and accessory genomes of *Psychrobacillus* strains using R (https://www.r-project.org/). Gene differences between strain PB01 and the five *Psychrobacillus* strains were visualized using the UpsetR package [18]. COG assignment of the core and unique genes of the six *Psychrobacillus* strains was carried out as described in the “Genome sequencing and annotation” section and were presented as a heatmap using R (v. 3.4.1). The TCA cycle, glyoxylate and anaplerotic pathways of *Psychrobacillus* sp. PB01 were in silico analyzed using the Pathway Tools v20.5 [19]. Phylogenetic trees were reconstructed using the neighbor-joining method [20].

## Results and Discussion

### Genome Analysis of Strain PB01

The genome of strain PB01 consists of a 4,332,095-bp circular chromosome with a G+C content of 36.0% (Fig. S1A) and one extrachromosomal circular plasmid (19,243 bp) with a G+C content of 34.3% (Fig. S1B). A total of 4,377 coding DNA sequences (CDSs) of the chromosome were predicted along with 33 rRNA and 77 tRNA genes (Table 1), resulting in a gene density of 1,036 genes/megabase. A total of 28 CDSs of the plasmid named ‘pPB01’ were predicted with a gene density of 1.56 genes/kilobase (Table 1). The identified 2,757 CDSs of the chromosome were classified into functional categories based on clusters of orthologous genes (COG) designation [21]. The classified genes are presented in the circular maps with color codes (Figs. S1A and S1B). The most abundant COG category of chromosome, except for [S] Function unknown (304 CDSs, 10.1%), was [R] General function prediction only (384 CDSs, 12.8%), followed by [E] Amino acid transport and metabolism (313 CDSs, 10.4%), [K] Transcription (227 CDSs, 7.6%), and [L] Replication, recombination and repair (223 CDSs, 7.4%), accounting for 38.1% of the overall CDS (1,147/3,007 CDSs)(Table S1). Generally, cold environments place physico-chemical stresses, such as low water activity, excessive UV or radiation, low solute diffusion, and low nutrient availability, on psychrophiles/psychrotolerants [1, 22]. To cope with unfavorable environments psychrophiles/psychrotolerants have evolved adaptive mechanisms by changing their genome content to gain high capacity for DNA repair, translation, and membrane transport [22]. Therefore, the high abundance of the COG categories might play important roles in the adaptation of strain PB01 to unique geographical locations (iceberg) [23].

### Comparative Genome Analysis of *Psychrobacillus* Strains

The general chromosomal features of strain PB01 were compared to the published chromosomes of the five *Psychrobacillus* strains (Table S2). The genomic G+C content of all six *Psychrobacillus* strains was 35.8-37.0%, which is within the reported range for *Psychrobacillus* strains. The chromosome sizes of all six *Psychrobacillus* strains were between 3.61 and 4.39 Mb, suggesting extensive gene gain and loss in the genus during evolutionary events. High variations in genome size were also observed in some psychrophile Psychrobacter strains [24]. Notably, the chromosome of strain PB01 encodes a higher number of copies of rRNA and tRNA genes (33 and 77) than any of the other five strains (Table S2). A high level of redundant rRNA and tRNA genes on genomes of psychrophiles is one of the survival strategies at low temperatures [25]. As such, the accelerated translation of special proteins caused by multiple copies of rRNA and tRNA genes may facilitate the fast cellular responses of strain PB01 to environmental changes, including rapid temperature shifts [26-28].

Average nucleotide identity (ANI) analysis helps elucidate the relationship between two strains via the identity/similarity values of the homologous regions of two genomes [29]. Thus, to obtain information on the lineage of strain PB01, compared to that of the five related strains, ANI analysis was performed using pyani with three alignment algorithms: a mummer (ANIm), blastn (ANIb), and blastall (ANIblastall), and alignment-free algorithm tetranucleotide signature frequencies (TETRA) (https://github.com/widdowquinn/pyani). The ANI values of the strain PB01 chromosome, compared against the genomes of the five related strains, were 0.836–0.866 with ANIm, 0.763–0.801 with ANIb, 0.758–0.795 with ANIblastall, and 0.982–0.991 with TETRA (Fig. S3), suggesting strain PB01 as a new species, in the genus *Psychrobacillus*, based on the commonly used ANIm, ANIb, and ANIblastall threshold values (< 0.95–0.96) [29], and the TETRA threshold value (< 0.99) for species delineation [30].

Following ANI calculation, synteny linkage of strain PB01 against the five related strains was further analyzed using progressiveMauve. As the genomes of the five reference strains were fragmented (Table S2), the contigs of the genomes were first reordered against the complete genome of strain PB01 with Mauve Contig Mover, following which synteny blocks (locally collinear blocks [LCB]) were analyzed using progressiveMauve with the recommended parameters (http://darlinglab.org/mauve/user-guide/progressivemauve.html). As inferred from the ANI analysis results, several large-scale synteny blocks (or LCB) were identified between the genomes of strain PB01 and the five related strains (Fig. S2A), reflecting a high degree of conservation among *Psychrobacillus* genomes. Outgoing link size in 5-kb windows and outgoing link number in 5-kb windows were shown in synteny linkages between the genomes of strain PB01 and the five related strains (Fig. S2B).

### Pan-Genome Analysis of *Psychrobacillus* Strains

**Fig. 1:**
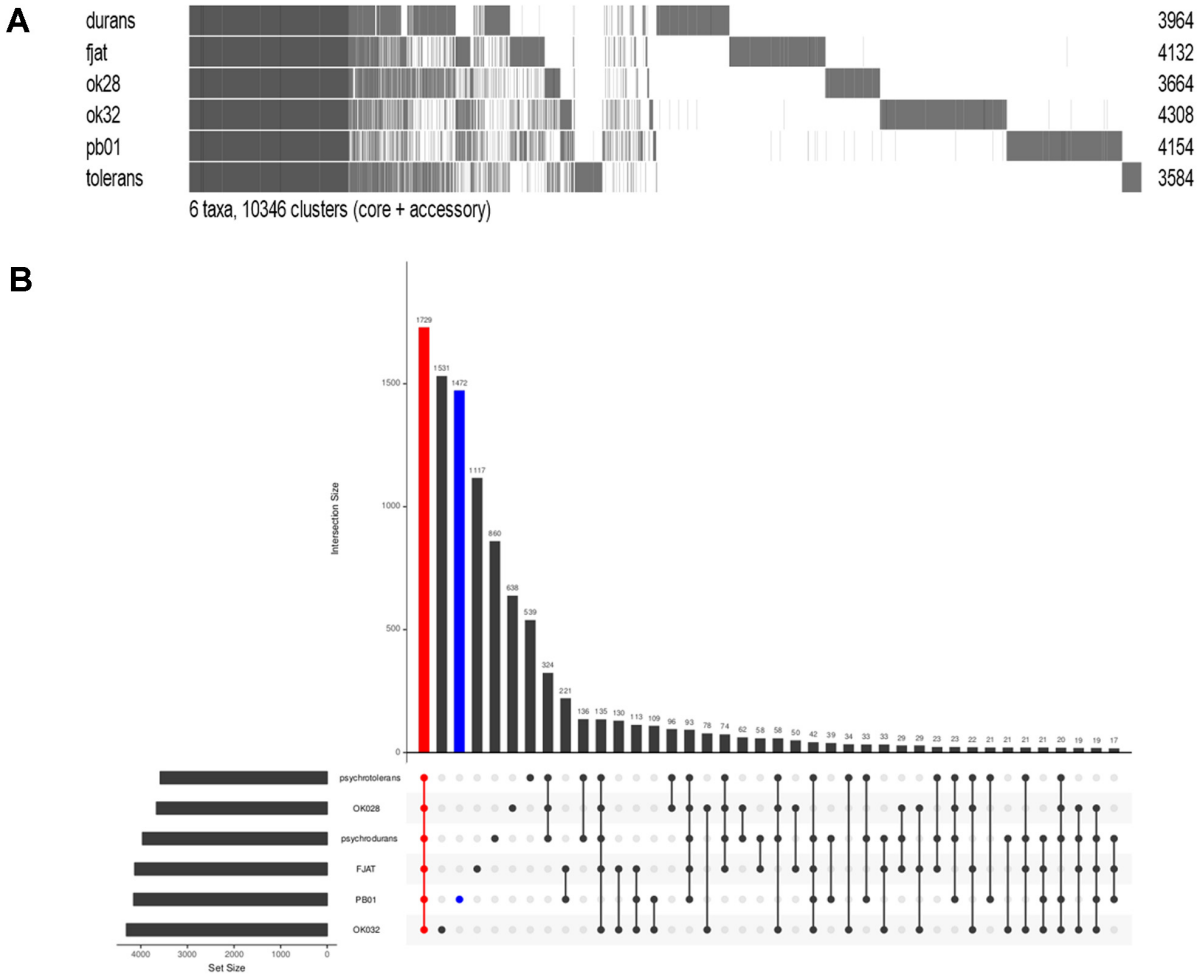
Pan-genome analysis of *Psychrobacillus* sp. PB01 and five related *Psychrobacillus* strains. (**A**) A gene presence/absence matrix plot generated using Roary. The numbers on the right represent the total number of core and unique genes of each *Psychrobacillus* strain. (**B**) Gene sharing among the *Psychrobacillus* sp. PB01 and the five other representative strains of *Psychrobacillus*. The bars represent the number (or size) of each intersection (dark circles) shown in the combination matrix. A red bar represents the number of core genes of the six *Psychrobacillus* strains; a blue bar highlights the number of unique genes of *Psychrobacillus* sp. PB01. The plot was drawn using UpSetR. Durans, *P. psychrodurans* DSM 11713; FJAT, *Psychrobacillus* sp. FJAT-21963; OK28, *Psychrobacillus* sp. OK028; OK32, *Psychrobacillus* sp. OK032; PB01, *Psychrobacillus* sp. PB01; Tolerans, *P. psychrotolerans*.

**Fig. 2:**
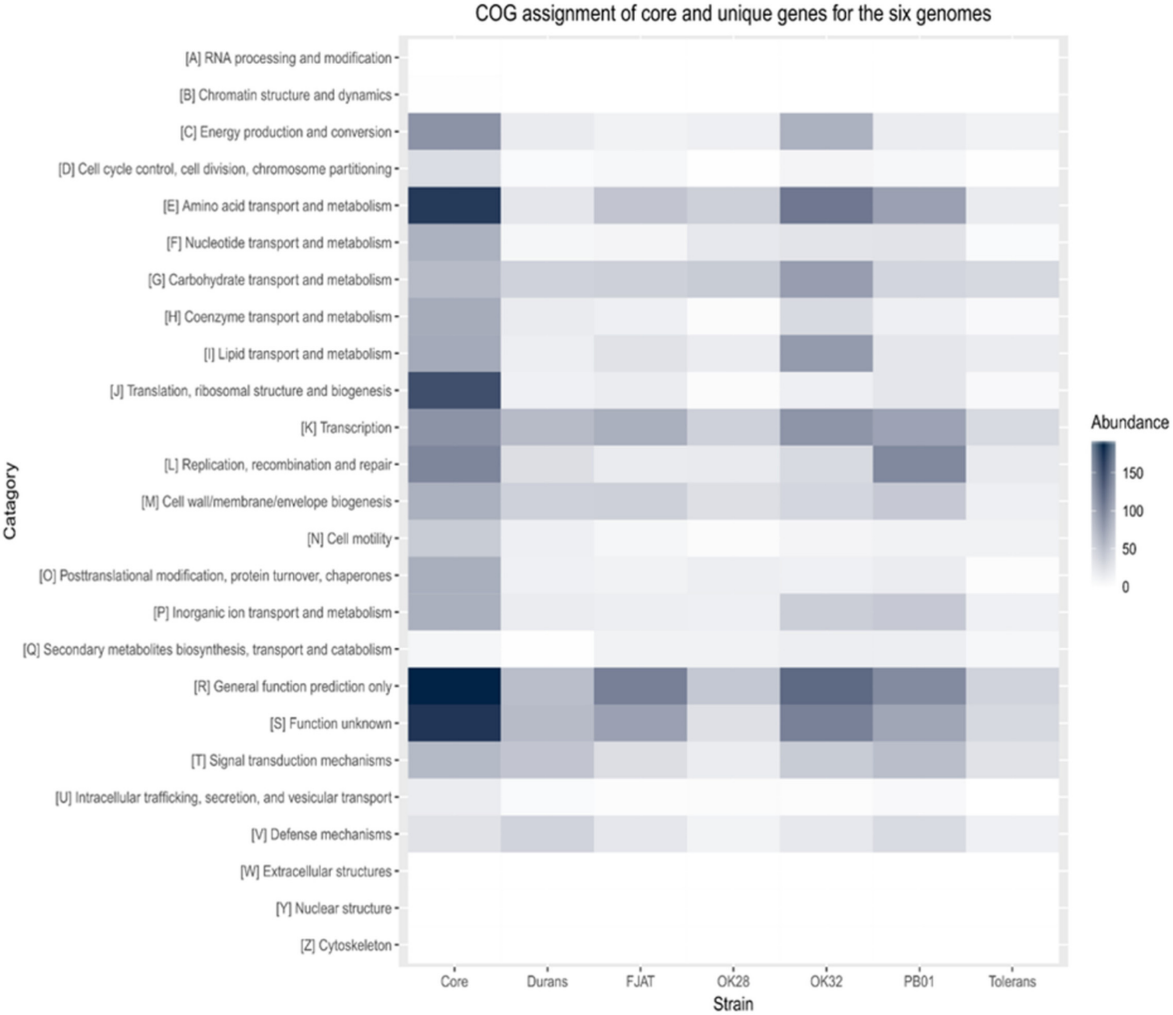
COG assignment of core and unique genes of the six *Psychrobacillus* strains. The comparative COG assignments of the core and unique genes of the six *Psychrobacillus* strains, obtained using Roary, were presented through a heatmap. Core, the core genes of the six *Psychrobacillus* strains; Durans, the unique genes of *P. psychrodurans* DSM 11713; FJAT, the unique genes of *Psychrobacillus* sp. FJAT-21963; OK28, the unique genes of *Psychrobacillus* sp. OK028; OK32, the unique genes of *Psychrobacillus* sp. OK032; PB01, the unique genes of *Psychrobacillus* sp. PB01; Tolerans, the unique genes of *P. psychrotolerans*. Scale of abundance (the number of genes) was represented by a continuous color gradient.

The pan-genome consists of the total number of genes of a species and can be used to obtain evolutionary event information for that species, including gene loss and gain [31]. Using Roary with a 70% minimum BLASTp identity [32], the pan-genomes of strain PB01 and the five *Psychrobacillus* strains were analyzed to determine the variations in gene content (Fig. 1). The pan-genome structure of the six strains comprises a total of 10,346 genes (Fig. 1A): 1,729 core genes (99% <= strains <= 100%) and 8,617 shell genes (15% <= strains < 95%). There are no soft core (95% <= strains < 99%) or cloud (0% <= strains < 15%) genes. The highest number of unique (or accessory) genes present in the *Psychrobacillus* strains (Fig. 1B) is 1,531 (OK032), followed by 1,472 (PB01), 1,117 (FJAT), 860 (pyschrodurans), 638 (OK028), and 539 (psychrotolerans). Unique genes of a species are usually involved in niche-specific adaptation [32]. The number of unique genes of each strain are related to the genome size of each *Psychrobacillus* strain (Table S2). The COG assignments of the core and unique genes of the six *Psychrobacillus* strains, including strain PB01, were analyzed to gain an insight into the difference between the gene functions of the strains (Fig. 2). Among the 1,729 core genes of the six strains, 1,513 genes were assigned into COG categories. The most abundant COG category of the core genes, except for [R] General function prediction only (187 genes, 12.4%) and [S] Function unknown (168 genes, 11.1%), was [E] Amino acid transport and metabolism (165 genes, 10.9%) and [J] Translation, ribosomal structure and biogenesis (144 genes, 9.5%). Notably, the unique genes assigned to [L] Replication, recombination and repair (93 genes), [T] Signal transduction mechanisms (50 genes), [P] Inorganic ion transport and metabolism (41 genes), and [J] Translation, ribosomal structure and biogenesis (19 genes) in strain PB01 were more abundant than those of the other five strains. As potential low-temperature adaptation strategies [23] strain PB01 has four genes encoding cold-shock proteins, two genes encoding DEAD-box RNA helicases, and eight genes encoding transporters for glycine betaine, which can serve as a cryoprotectant [33], on the genome.

### In Silico Analysis of TCA Cycle and Glyoxylate Bypass of *Psychrobacillus* Strains

**Fig. 3:**
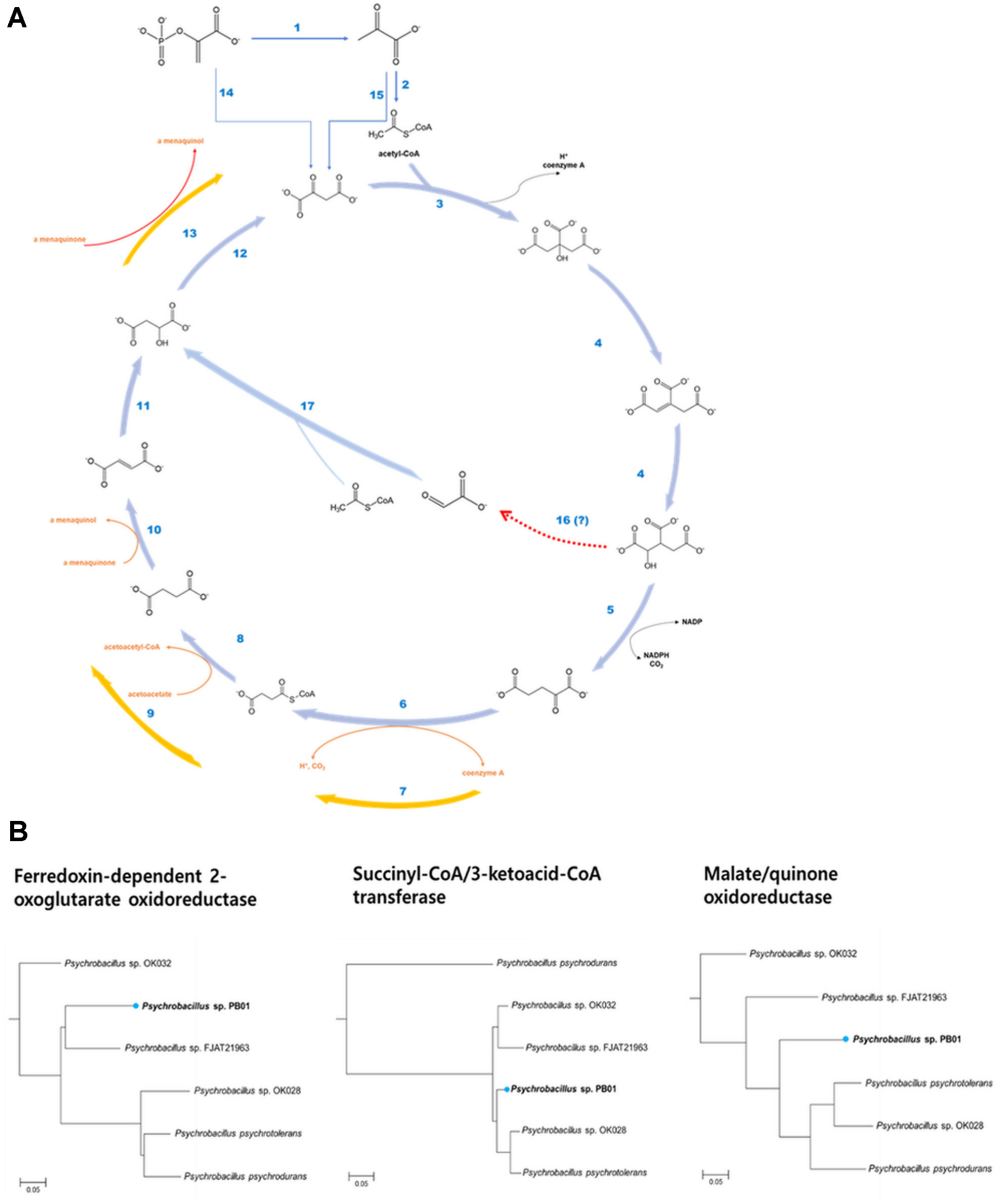
In silico prediction of TCA cycle, glyoxylate bypass, and anaplerotic pathway of *Psychrobacillus* sp. PB01 (**A**) and phylogenetic trees of ferredoxin-dependent 2-oxoglutarate oxidoreductase, succinyl-CoA/3-ketoacid CoA transferase, and malate/quinone oxidoreductase of *Psychrobacillus* strains (**B**). (**A**) The TCA cycle, glyoxylate and anaplerotic pathways of *Psychrobacillus* sp. PB01 were in silico analyzed using the Pathway Tools. Additional three reactions (7, 9, and 13) of the TCA cycle were represented by yellow arrows. The pathway enzymes involved in reactions are (1) Pyruvatekinase, (2) Pyruvate dehydrogenase, (3) Citrate synthase, (4) Aconitase, (5) Isocitrate dehydrogenase, (6) NAD+-dependent 2- oxoglutarate dehydrogenase, (7) ferredoxin-dependent 2-oxoglutarate oxidoreductase, (8) Succinyl-CoA ligase, (9) Succinyl-CoA/3-ketoacid CoA transferase, (10) Succinate dehydrogenase, (11) Fumarate hydratase, (12) Malate dehydrogenase, (13) Malate/quinone oxidoreductase, (14) Phosphoenolpyruvate carboxykinase, (15) Pyruvate carboxylase, (16) a missing enzyme, and (17) Malate synthase. (**B**) Phylogenetic trees of ferredoxin-dependent 2-oxoglutarate oxidoreductase (subunit alpha), succinyl-CoA/3-ketoacid CoA transferase (subunit A), and malate/quinone oxidoreductase of six *Psychrobacillus* strains were obtained using the neighbor-joining method. Bar, 0.05 substitutions per amino acid position.

In silico pathway analysis uncovered that the genome of strain PB01 has genes encoding all of the enzymes involved in the TCA cycle (Fig. 3A), suggesting that the TCA cycle of strain PB01 would be fully operative in the oxidative direction [34]. Notably, in addition to the complete TCA cycle enzymes, additional genes encoding three enzymes were annotated as ferredoxin-dependent 2-oxoglutarate synthase, succinyl-CoA/ acetoacetyl-CoA transferase, and malate/quinone oxidoreductase. These three enzymes had been reported as alternative enzymes of the TCA cycle in *Helicobacter pylori* [35]. It remains a question as to whether two types of the enzymes would be fully active simultaneously and function differently under physiological or environmental conditions. As suggested in the previous study [36], it is possible that two types of enzymes would function differently to meet metabolic needs in the cellular carbon metabolism. Comparative genome analysis revealed that the genomes of the other *Psychrobacillus* strains also encode the three enzymes (Fig. 3B). Thus, the observed co-existence of TCA cycle enzymes would be unique in *Psychrobacillus* strains. The anaplerotic formation of oxaloacetate from phosphoenolpyruvate (PEP) and pyruvate was predicted by the presence of genes encoding PEP carboxykinase and pyruvate carboxylase, respectively (Fig. 3A). However, there is no candidate gene encoding PEP carboxylase, which is the third anaplerotic enzyme, in the genome of strain PB01. Among two glyoxylate pathway enzymes (isocitrate lyase and malate synthase) only one gene encoding malate synthase was identified. There are no homologs of isocitrate lyase genes in the genome of strain PB01.

In summary, the complete genome of strain PB01 consists of a single circular chromosome and a plasmid. Comparative genomic analysis suggests extensive gene gain and loss in the genus during evolutionary events. The pan-genome of the six strains analyzed comprises 1,729 core genes and 8,617 unique (accessory) genes. An abundance of unique genes assigned to (i) Replication, recombination, and repair,(ii) Signal transduction mechanisms, (iii) Inorganic ion transport and metabolism, and (iv) Translation, ribosomal structure, and biogenesis in the chromosome of strain PB01 suggests that they play critical roles in the adaptation of this strain to extreme environments. Notably, *Psychrobacillus* strains possess a complete TCA cycle as well as three additional *H. pylori*-type enzymes. The co-presence of the genes for TCA cycle enzymes could facilitate the research for variation and evolution of the TCA cycle.

## Supplemental Materials



Supplementary data for this paper are available on-line only at http://jmb.or.kr.

## Figures and Tables

**Table 1:** General genome features of *Psychrobacillus* strain PB01.

Attribute	Chromosome	Plasmid	Total
Genome size (bp)	4,332,095	19,243	4,351,338
Number of contigs	1	1	2
G+C content (%)	36.0	34.3	36
CDS	4,377	28	4,405
rRNA (5S, 16S, 23S)	33	0	33
tRNA	77	0	77
