# Supplementary material for: Comparative Genome Analysis of Psychrobacillus Strain PB01, Isolated from an Iceberg
Source: J Microbiol Biotechnol. 2019 Dec 9;30(2):237–43. doi: 10.4014/jmb.1909.09008 (PMC9728334; doi:10.4014/jmb.1909.09008)

J. Microbiol. Biotechnol.
https://doi.org/10.4014/jmb.1909.09008

jmb

Table S1. COG stat of genome features of strain PB01

[1]COG code	Function category	Number of [2]CDS
		Chromosome
B	Chromatin structure and dynamics	1
C	Energy production and conversion	128
D	Cell cycle control, cell division, chromosome partitioning	37
E	Amino acid transport and metabolism	313
F	Nucleotide transport and metabolism	106
G	Carbohydrate transport and metabolism	155
H	Coenzyme transport and metabolism	96
I	Lipid transport and metabolism	118
J	Translation, ribosomal structure, and biogenesis	189
K	Transcription	227
L	Replication, recombination, and repair	223
M	Cell wall/membrane/envelope biogenesis	156
N	Cell motility	70
O	Posttranslational modification, protein turnover, and chaperones	100
P	Inorganic ion transport and metabolism	141
Q	Secondary metabolites biosynthesis, transport, and catabolism	29
R	General function prediction only	384
S	Function unknown	304
T	Signal transduction mechanisms	138
U	Intracellular trafficking, secretion, and vesicular transport	24
V	Defense mechanisms	68

[1] COG: Clusters of Orthologous Groups

[2] CDS: Coding Sequence

Table S2. A comparison of chromosome features between strain PB01 and five related *Psychrobacillus* strains

	**Strain PB01**	***P. psychrotolerans* DSM 11706**	***P. psychrodurans* DSM 11713**	***Psychrobacillus* sp. FJAT-21963**	***Psychrobacillus* sp. OK032**	***Psychrobacillus* sp. OK028**
Size (Mb)	4.33	3.61	4.02	4.10	4.39	3.76
Number of contigs (Scaffolds)	1	21 (20)	48 (48)	64 (43)	41 (41)	21 (21)
G+C content (%)	36.0	36.4	36.0	35.8	37.0	36.6
CDS	4,377	3,511	3,902	3,990	4166	3,588
rRNA	33	23	22	14	25	18
tRNA	77	55	61	62	68	54
Accession	CP031223	NZ_FOXU00000000.1	NZ_FOUN00000000.1	NZ_LJIY00000000.1	NZ_FOGY00000000.1	NZ_FNHY00000000.1

Fig. S1. Circular representation of chromosome (A) and plasmid (B) genomes of *Psychrobacillus* sp. PB01. (A) From the outer circle to the inner circle: predicted protein-coding sequences (colored by COG categories) on the plus strand, predicted protein-coding sequences (colored by COG categories) on the minus strand, RNA genes (tRNAs, blue; rRNAs, red), GC content (blue/black), and GC skew (red/black). (B) From the outer circle to the inner circle: predicted protein-coding sequences (colored by COG categories) on the plus strand, predicted protein-coding sequences (colored by COG categories) on the minus strand, GC content (blue/black), and GC skew (red/black).

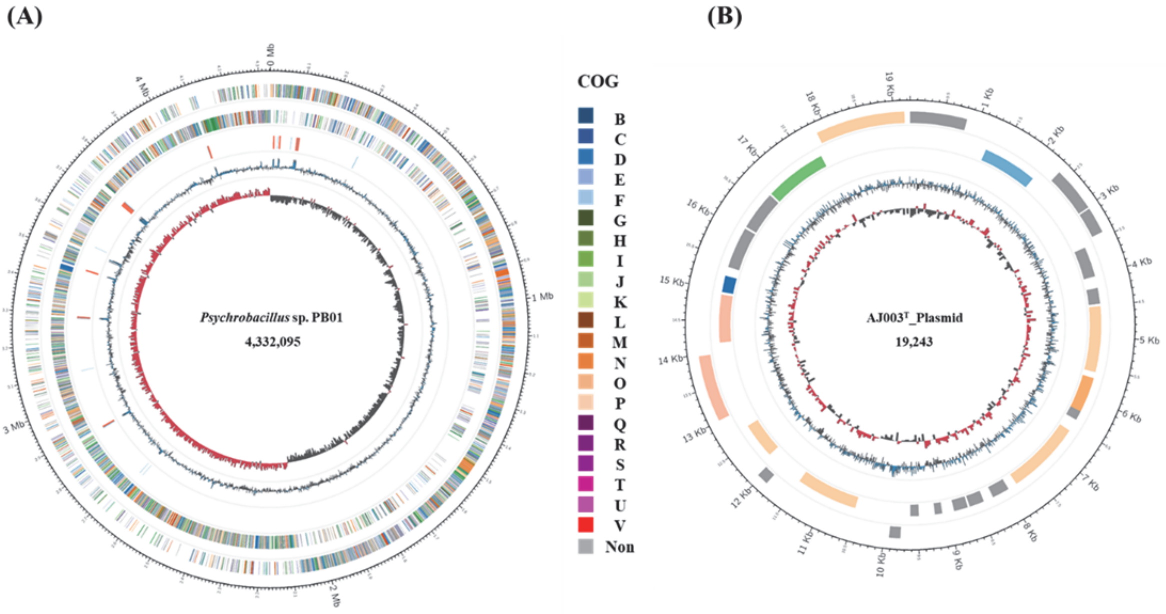

Fig. S2. Synteny blocks (A) and synteny linkages (B) between the genomes of *Psychrobacillus* sp. PB01 and five related *Psychrobacillus* strains. (A) The color blocks, which were generated using progressiveMauve, represent the synteny blocks (locally collinear blocks) of the genomes of *Psychrobacillus* sp. PB01 and the five related *Psychrobacillus* strains. (B) Color synteny linkages were generated using Circos v6.7. The ideograms on the left represent equal proportions of the genomes of five related *Psychrobacillus* strains (*P. psychrotolerans* DSM 11706: blue; *P. psychrodurans* DSM 11713: green; *Psychrobacillus* sp. OK032: yellow; *Psychrobacillus* sp. OK028: orange; *Psychrobacillus* sp. FJAT-21963: red). The inner histogram and outer stacked histogram on the right (ideogram of the *Psychrobacillus* sp. PB01 genome (black)) represent outgoing link size in 5-kb windows and outgoing link number in 5-kb windows, respectively.

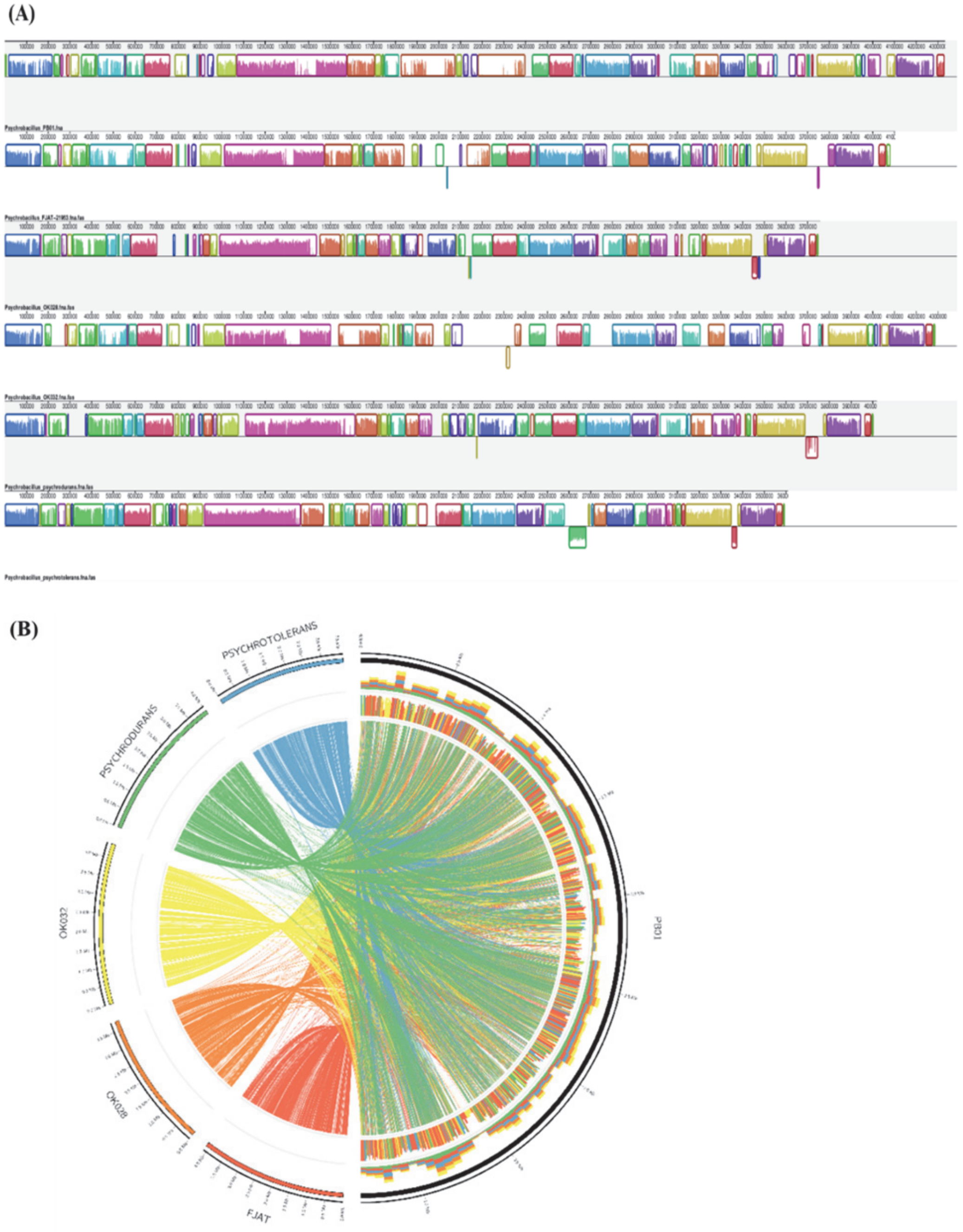

Fig. S3. Average nucleotide identity (ANI) analysis of chromosomes of *Psychrobacillus* sp. PB01 and five related *Psychrobacillus* strains. ANI analysis was performed using pyani with ANIm (A), ANIb (B), ANIblastall (C), and TETRA (D) algorithm. ANI similarity values (%) are indicated in the heatmaps. Scale of similarity values are represented by a continuous color gradient.

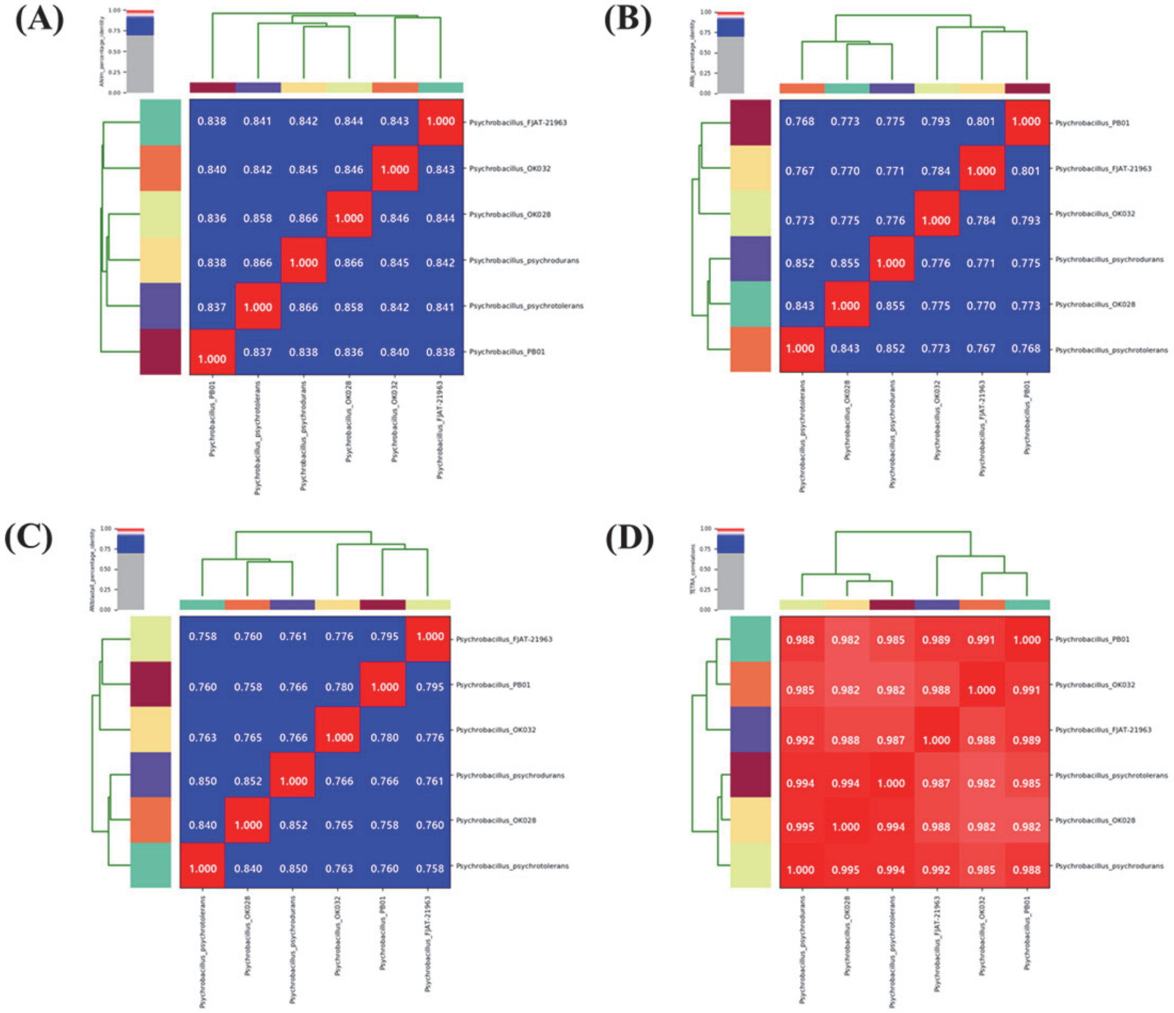

Supplement: Supplementary file 1 [file JMB-30-2-237-supple.pdf]
